# Local Anodizing of a Newly Prepared Aluminum Micrometric Disk

**DOI:** 10.3390/nano12050845

**Published:** 2022-03-02

**Authors:** Ludovic Cicutto, Jérome Roche, Laurent Arurault

**Affiliations:** CIRIMAT, Université de Toulouse, CNRS, UT3 Paul Sabatier, Bât. CIRIMAT, 118 Route de Narbonne, CEDEX 9, 31062 Toulouse, France; ludoviccicutto@orange.fr (L.C.); jerome.roche@univ-tlse3.fr (J.R.)

**Keywords:** local anodizing, aluminum, micrometric disk, expansion factor, film growth rate

## Abstract

A search through the literature reveals that the vast majority of studies about aluminum anodizing were conducted at the macroscale (i.e., from cm^2^ up to m^2^), while those focused on local anodizing (i.e., on surfaces of less than 1 mm^2^) are rare. The last ones either used insulating masks or were conducted in an electrolyte droplet. The present study describes on the one hand a new way to prepare aluminum microelectrodes of conventional disk-shaped geometry, and on the other hand the local anodizing of their respective aluminum micrometric top-disks. The influence of the anodizing voltage on anodic film characteristics (i.e., thickness, growth rate and expansion factor) was studied during local anodizing. Compared with the values reported for macroscopic anodizing, the pore diameter appears to be significantly low and the film growth rate can reach atypically high values, both specificities probably resulting from a very limited increase in the temperature on the aluminum surface during anodizing.

## 1. Introduction

The anodizing of aluminum and its alloys has been extensively studied since it was first reported by Buff and coworkers in 1857 [1]. At the end of the 19th century, the mass production of aluminum began, largely using the Hall–Heroult reduction process. Aluminum anodizing was extensively studied throughout the 20th century up to the present day. However, the vast majority of those studies were conducted for heavy industrial purposes at the macroscale, i.e., on surfaces of between a few cm^2^ up to a few m^2^. Studies of aluminum anodizing at the microscale (local anodizing), i.e., on surfaces of less than 1 mm^2^, are rare. However, studies are currently underway to develop new applications of local anodizing in different fields, such as nanointerconnections [2], metal multicore microelectrodes [3] and the low-cost fabrication of micropatterned comet assay for DNA damage quantification [4].

Most local anodizing methods that do exist can be classified into two categories. The first includes the local anodizing of aluminum conducted using different types of insulating mask on a macroscopic substrate: a polymer film [5], a self-assembled monolayer of polystyrene microspheres [6] or silicon oxide [2]. Techniques under the second category use small electrochemical probes as cathodes to concentrate the current on small areas of a macroscopic aluminum substrate. However, due to the insulating nature of an anodic film, conventional local electrochemical techniques, such as SECM (scanning electrochemical microscopy), cannot be applied as they lead to the scattering of the current as the film grows. To avoid this issue [7,8,9], local anodizing is usually conducted in an electrolyte droplet. However, both approaches use macroscopic aluminum surfaces onto which a small anodic film is added.

The aim of the present study is to achieve, for the first time, a microscale anodic film on an aluminum surface with overall micrometric dimensions. For this purpose, an innovative method to fabricate aluminum microelectrodes of conventional disk-shaped geometry with overall micrometric dimensions was proposed. Each aluminum microelectrode was subsequently anodized in a conventional sulfuric acid electrolyte to produce a porous anodic oxide film. This study finally analyzed the specificities of the anodic film characteristics (i.e., thickness, growth rate and expansion factor) for local anodizing compared with the values reported for macroscopic anodizing.

## 2. Materials and Methods

### 2.1. Innovating Fabrication of Disk-Shaped Aluminum Microelectrode

**Figure 1:**
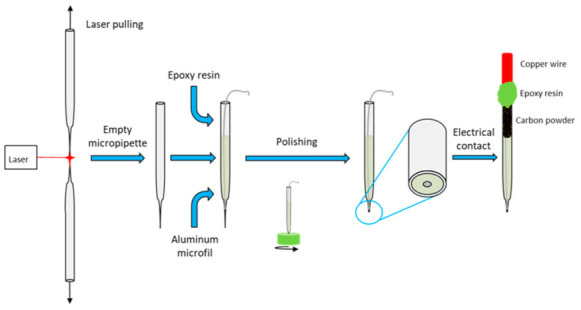
Synoptic diagram of the new preparation of the aluminum micrometric disk.

Specific issues for the preparation of disk-shaped aluminum microelectrodes are explained in Appendix A. An innovative method is proposed here for the first time. Borosilicate glass capillaries with an inner diameter of 0.69 mm, an outer diameter of 1.2 mm and a length of 10 cm were supplied by Corning (Corning, NY, USA). They were locally heated and simultaneously pulled using a laser-based automatic puller P-2000 (Sutter Instrument, Novato, CA, USA). Unlike conventional methods, the capillaries were pulled without Al microwire to avoid melting and oxidation of the metal (Figure 1). The parameters adopted for the pre-programmed pulling steps (Heat = 250, Fil = 0, Vel = 80, Del = 128 and Pul = 50) were chosen to obtain capillaries with reproducible shapes and sufficient glass wall thickness at the tip to allow for the subsequent insertion of Al microwires without the risk of breakage. Al 1050 microwires of 99.5% purity and 125 µm diameter were procured from Goodfellow (Lille, France).

An epoxy resin (Presi MA2+, Eybens, France) was injected into the pre-pulled capillary and an Al microwire was inserted up to the small diameter tip. The epoxy resin was then left to cure at room temperature for 24 h. Prior to the polishing step, the capillary was molded into an epoxy resin pellet of 1.5 cm in diameter and 1 cm in thickness to facilitate the polishing of the microelectrode. This specific resin was supplied by Presi (KM Back, Eybens, France) and chosen for its solubility in acetone in order to dissolve the pellet after polishing. The microelectrode tip was gently and manually polished using rotating disks with SiC particles of sequentially reduced grain size (P600, P1200, P2400 and P4000). A second polishing step was performed using an aqueous 0.25 µm alumina suspension on the polishing pad. The epoxy resin pellet was then dissolved in acetone. A conductive carbon black powder was used to fill the inside of the capillary to ensure electrical contact between the Al microwire and a 0.5 mm diameter copper wire inserted to the rear of the capillary. The copper wire and the capillary were sealed using epoxy resin. The copper wire was then used to connect the disk-shaped microelectrode to the generator for the anodizing step.

**Figure 2:**
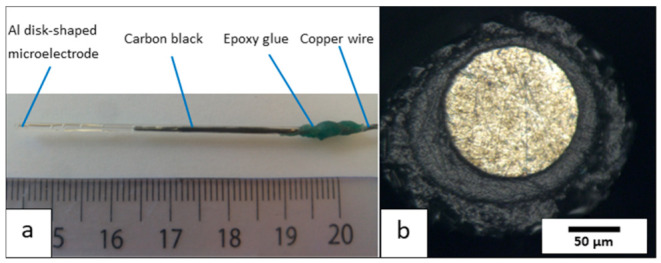
(**a**) Photo of the aluminum microelectrode system and (**b**) optical view of its surface.

The resulting disk-shaped aluminum microelectrode system is shown in Figure 2a.

This new experimental protocol makes it possible to repeatedly fabricate 1050 aluminum microelectrodes of the disk-plane type with an active surface area of about 125 μm in diameter. The ratio (called Rg) between the total radius of the electrode and the radius of the active surface can vary between 1.5 and 9.5 (Figure 2b). Furthermore, when the electrical resistance was experimentally measured for these prepared electrodes, i.e., between the external copper contact and the surface of the aluminum disk, the resistance of the “copper wire/carbon paste/aluminum microwire” electrochemical chain was 0.9 Ohm, i.e., a low value.

### 2.2. Local Anodizing and Subsequent Characterizations

**Figure 3:**
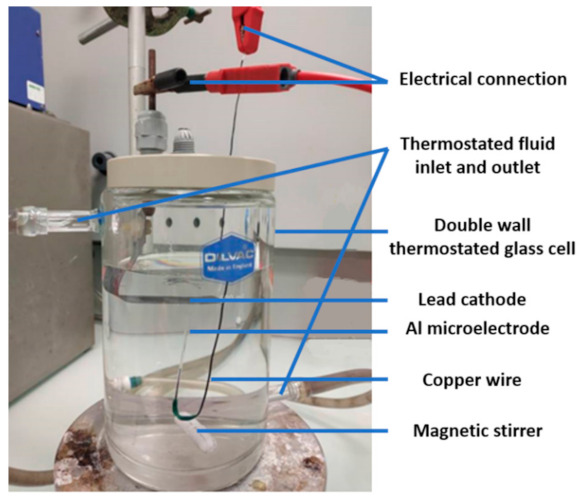
Photo of the anodizing cell.

Prior to anodizing, the Al microelectrode was chemically etched in 25 g·L^−1^ NaOH solution at 40 °C for 2 min and then washed with deionized water. A desmutting step was then performed in a 25% *v/v* nitric acid solution at room temperature for 2 min. Finally, the microelectrode was thoroughly rinsed with deionized water and dried. Anodizing was carried out in a thermostatically controlled cell at 20 °C (Figure 3).

A lead plate was used as a cathode and the Al microelectrode was placed vertically with the disk-shaped electrode facing the air/electrolyte interface. This orientation was chosen to facilitate the evacuation of oxygen bubbles generated during anodizing that could induce electrical insulation of the microelectrode tip. Anodizing was conducted in a one-step process at a constant voltage (from 20 to 90 V) for 20 min using a Keithley 2611a (Beaverton, OR, USA) generator. The electrolyte was a 0.15 M sulfuric acid solution, stirred using a magnetic stirrer at 300 rpm.

Three-dimensional optical microscopy (3D Keyence, Bois-Colombes, France) and scanning electron microscopy with a field emission gun (FEG-SEM JEOL JSM6700F, Croissy-sur-Seine, France) were both used to characterize the resulting porous anodic films. Then, porosity was characterized using binarization and ImageJ software.

## 3. Results

### 3.1. Influence of the Anodizing Voltage

**Figure 4:**
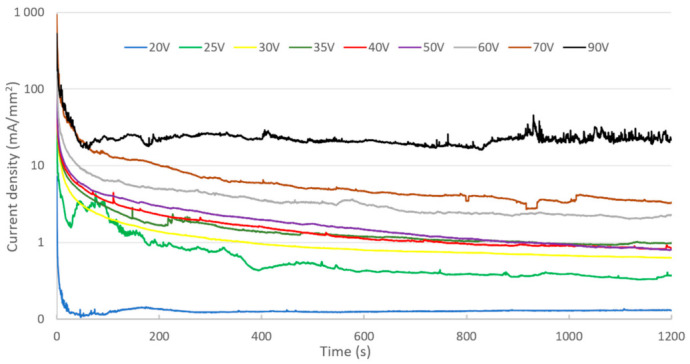
Evolution of current density for different cell voltages in sulfuric acid electrolyte (0.15 M).

Figure 4 shows the evolution of current density during anodizing performed at a constant voltage ranging from 20 to 90 V.

There are two types of current density evolution. For a voltage of 20 V, a decrease in current, followed by a slight increase, is observed. After reaching a peak, the current then decreases to a low and constant value. For higher voltages (25–90 V), the change in current density as a function of time shows an exponential-type decrease. In the first moments after switching on the cell, a peak in current is recorded, its value increasing with cell voltage. The monotonous decrease in the current has a similar shape, whatever the value of the applied voltage. On the other hand, the value of the current at the end of anodizing is proportional to the applied cell voltage.

**Figure 5:**
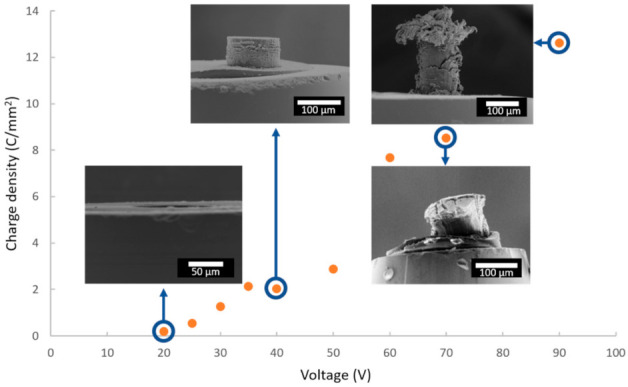
Evolution of the charge density as a function of the anodizing voltage, with FEG-SEM images for voltages of 20, 40, 70 and 90 V.

Since the duration of anodization is the same (i.e., 1200 s) for all samples, the total charge densities can be compared, depending on the applied voltage. Figure 5 shows the quantity of total charges exchanged over the entire duration of the anodizing as a function of the cell voltage, with FEG-SEM images of some corresponding anodic films.

**Figure 6:**
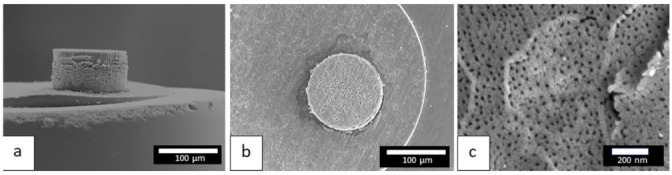
FEG-SEM images of the anodic film prepared at 40 V, (**a**) side view, (**b**) global surface view, (**c**) detailed surface view.

The more the cell voltage increases, the more the quantity of charges increases, which induces a thicker anodic film. However, above a voltage of 50 V, the anodic films present numerous cracks, or even burst, making it impossible to accurately measure the characteristics of these anodic films. On the other hand, Figure 6a shows a side view of the anodic film prepared at 40 V, while Figure 6b,c show global and detailed surface views, respectively.

In particular, the detailed FEG-SEM surface view (Figure 6c) shows the emerging porosity. Using binarization and ImageJ software, this porosity was then characterized assuming circular pores. For the anodic film prepared at 40 V, the mean pore diameter was found to be 17 nm, while the mean interpore distance was 51 nm. Furthermore, the enlargement rate of pore diameter was estimated to be about 0.2 nm/V in the 25–90 voltage range.

### 3.2. Measurement of the Expansion Factor

For anodic films prepared at voltages higher than 50 V, it was impossible to accurately measure the height of the film, and therefore to calculate the expansion factor, also known as the Pilling–Bedworth ratio (PBR). This factor corresponds to the ratio between the molar volume of the anodic film and the molar volume of the electrochemically converted metal; it is usually estimated by measuring the ratio between the height of the formed anodic film and the height of the initial metal converted by oxidation [10,11,12]. In the present case, this approximation is all the more valid as the radial expansion is very low or tends to zero. Indeed, the anodic film starts to grow at the aluminium surface, and about 2/3 of the growing anodic film is constrained radially by the presence of the surrounding epoxy resin (Presi MA2+).

Experimentally, since the anodic film was partially present under the surface of the electrode, its thickness was measured twice using a 3D optical microscope. A first observation made it possible to determine the external height of the anodic film, relative to the surface of the electrode. The anodic film was then immersed (2 h) in an aqueous solution (at 40 °C) containing 0.64 M of phosphoric acid and 0.15 M of chromium trioxide, allowing it to dissolve selectively, while leaving the aluminum substrate intact (Appendix B). Once dissolved, the electrode was again observed under a 3D optical microscope, and the height difference between the surface of the aluminum substrate and the surface of the electrode corresponded to the thickness of the metal consumed, allowing the experimental value of the PBR to be obtained.

**Figure 7:**
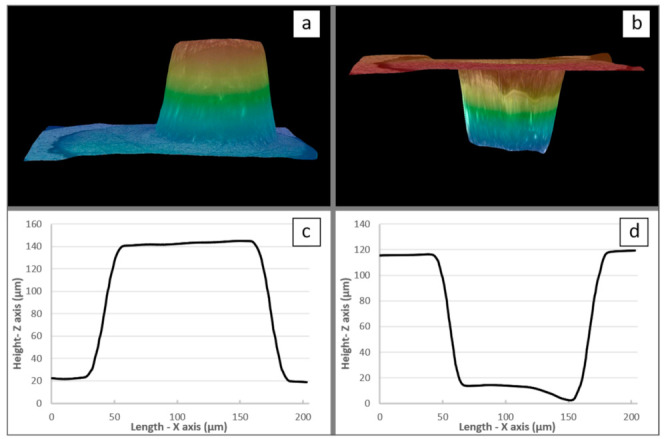
Three-dimensional images (**a**) before and (**b**) after dissolution of the film obtained at 50 V and the corresponding 2D images, i.e., (**c**) before and (**d**) after dissolution.

Accordingly, given the alteration of the anodic film at higher voltage values, the experimental PBR measurement was performed for voltages equal to or less than 50 V. For instance, Figure 7a,b, show 3D optical views before and after chemical dissolution of the anodic film, respectively. In this case (i.e., at 50 V as applied voltage), the oxide film thickness was found to be 218 µm, while the expansion factor (PBR) was equal to 2.10. Table 1 shows a complete set of results for the 20–50 V voltage range.

## 4. Discussion

First of all, FEG-SEM observations corroborate the interpretations made with regard to the evolution of the current density for the various values of the applied voltage. Below a value of 25 V, there is in fact no growth of an anodic film, while the partial destruction of the anodic films occurs for voltages above 60 V.

The first behavior (i.e., U ≤ 25 V) probably results from the predominance of the chemical dissolution of the anodic film during contact with the anodizing electrolyte, while the anodic polarization is too low to ensure sufficient growth of the anodic film.

For the second behavior (i.e., 30 < U < 50 V), effective undamaged porous anodic films are obtained. The oxide film thickness was found to be from 109 to 218 µm (Table 1), while the expansion factor (PBR) varied from 1.80 to 2.10, and the corresponding film growth rate was between 327 and 654 µm/h. Therefore, in the second case, the expansion factor seems to be high. However, the rigorous interpretation of an expansion factor value is difficult because it depends on many factors [10,11,12], such as the porosity of the anodic film, their hydration, and/or the inclusion of ions, e.g., here sulfate ions from the electrolyte.

For the third behavior (i.e., U ≥ 60 V), it could be due to several factors, in particular the chemical dissolution of the tallest films formed at the highest voltages, as well as the internal stresses of the film linked to high experimental values of the Pilling–Bedworth ratio. Alternatively, the expansion of anodic films causes friction between the epoxy resin and the anodic film, inducing stresses in both. The stresses of the film are released out of the microelectrode (Figure 5), which sometimes leads to its partial destruction, while the epoxy resin can partially come out of the microelectrode at high PBR values.

Moreover, voltage values (20–90 V) applied in this study may appear high with the voltage usually being lower than 25 V when anodizing is performed in a sulfuric acid-based electrolyte at 20 °C. In the present study, the voltage values are in fact more comparable to those used for hard anodizing (i.e., sulfuric acid anodizing at lower temperatures of about −5 or 0 °C) requiring higher voltage values of up to 100 V. The growth of the anodic film here is very rapid (327–654 µm/h) compared with usual film growth rates (about 10–70 µm/h) obtained on large aluminum surfaces [13,14]. These results could be explained by considering the specificities of local anodizing on a micrometric surface, especially both the electrolyte temperature and the aluminum superficial temperature, rigorously studied by Terryn’s team [15,16,17].

Local anodizing involves low electrical charges (e.g., 24.5 mC at 40 V for 1200 s) as well as high heat dissipation (considering the ratio between the electrode surface and the electrolyte volume), inducing a very limited temperature increase on the aluminum surface during anodizing, unlike anodizing performed on a large surface. Due to this fairly low temperature, the kinetics of the film dissolution (i.e., the main chemical reaction) is also low, limiting the pore enlargement to about 0.2 nm/V, i.e., significantly lower than the values reported for anodic films on macroscopic electrodes [18,19]. At the same time, the growth rate of anodic films should be slowed down because of the fairly low temperature on the aluminum surface. However, the growth rate mainly depends on the anodizing voltage. Here, medium and high voltages values (from 20 to 90 V) have been applied to perform local anodizing for 20 min at 20 °C (bulk and interfacial temperatures), ensuring a paradoxically high growth rate (327–654 µm/h). Therefore, local anodizing requires higher voltages (20–90 V) than those usually used (U < 25 V) for large surfaces in a sulfuric acid electrolyte thermoregulated at 20 °C.

The present work studied the influence of the anodizing voltage on anodic film characteristics during local anodizing on a newly prepared micrometric aluminum surface. However, voltage is only one of the operating parameters potentially influencing these characteristics. Other parameters (e.g., composition and temperature of the electrolyte) are known to have significant influences on the film characteristics and on its growth, and it would therefore be interesting to devote an extensive study to them in the case of local anodizing.

## Figures and Tables

**Table 1:** Thickness of the anodic film and corresponding expansion factor as a function of voltage.

Anodizing Voltage	Thickness (μm)	Expansion Factor (PBR)	Growth Rate (µm/h)
20	3	-	9
35	109	1.96	327
40	115	1.80	345
50	218	2.10	654

## Data Availability

Data presented in this article is available on request from the corresponding author.

## Appendix A

Specific issues for the preparation of disk-shaped aluminum microelectrodes:

A conventional approach to fabricate disk-shaped microelectrodes involves the local heating of a glass capillary containing a metal microwire followed by a pulling stage [20,21]. The capillary then breaks up along a plane orthogonal to the capillary axis and the metal microwire becomes sealed into the glass. After polishing, the active electrode surface is defined by the wire cross-section. Gold and platinum are the most frequently used metals and a survey of the literature indicates that this technique has not been applied to produce an aluminum microelectrode. This can be explained by the fact that unlike platinum and gold, aluminum has a melting point lower than the glass transition temperature of the borosilicate glass capillary (Table A1). Furthermore, aluminum has a much higher thermal expansion coefficient than the borosilicate glass capillary (Table A1), creating mechanical stresses in the system.

**Table A1:** Melting points and thermal expansion coefficients of metals used for disk-shaped microelectrodes.

Metal	Melting Point (°C)	Linear Thermal Expansion Coefficient (×10^7^ K^−1^)
Platinum	1768	90
Gold	1064	142
Silver	962	190
Aluminum	660	240
Borosilicate	821 (Tg)	35

Finally, when aluminum is heated under normal atmospheric conditions, a spontaneous and exothermic (−838 kJ·mol^−1^) oxidation reaction occurs according to Equation (A1).

2Al + 3/2O_2_ → Al_2_O_3_ (A1)

These physical properties of aluminum explain why the conventional glass capillary pulling method has not been reported for the fabrication of an aluminum microelectrode.

## Appendix B

Before carrying out the experimental measurements to evaluate the expansion factor, an unanodized sample was immersed in the chromo-phosphoric acid solution used to dissolve the anodic film. FEG-SEM observations of its surface, before and after immersion in the dissolving solution, show that the aluminum surface is unaltered and therefore that this acidic solution does not attack the metal under the time and temperature conditions used.
